# Monofluorophosphate Blocks Internal Polysaccharide Synthesis in *Streptococcus mutans*

**DOI:** 10.1371/journal.pone.0170483

**Published:** 2017-01-26

**Authors:** Ana M. Demonte, Matias D. Asencion Diez, Conrad Naleway, Alberto A. Iglesias, Miguel A. Ballicora

**Affiliations:** 1 Laboratorio de Enzimología Molecular, Instituto de Agrobiotecnología del Litoral (UNL-CONICET), Santa Fe, Argentina; 2 Department of Chemistry and Biochemistry, Loyola University Chicago, Chicago, Illinois, United States of America; University of Helsinki, FINLAND

## Abstract

*Streptococcus mutans* is the leading cause of dental caries worldwide by accumulating a glycogen-like internal polysaccharide (IPS) that contributes to cariogenicity when sugars are in excess. Sodium monofluorophosphate (MFP) is an active anticariogenic compound in toothpastes. Herein, we show that MFP inhibits (with an *I*_0.5_ of 1.5 mM) the *S*. *mutans* ADP-glucose pyrophosphorylase (EC 2.7.7.27), which catalyzes the key step in IPS biosynthesis. Enzyme inhibition by MFP is similar to orthophosphate (Pi), except that the effect caused by MFP is not reverted by fructose-1,6-bisP, as occurs with Pi. Inhibition was correlated with a decrease in acidogenesis and IPS accumulation in *S*. *mutans* cells cultured with 2 mM sodium MFP. These effects were not mimicked by sodium fluoride. Considering that glycogen synthesis occurs by different pathways in mammals and bacteria, ADP-glucose pyrophosphorylase could be visualized as a molecular target for controlling *S*. *mutans* virulence. Our results strongly suggest that MFP is a suitable compound to affect such a target, inducing an anticariogenic effect primarily by inhibiting a key step in IPS synthesis.

## Introduction

It has been demonstrated that *S*. *mutans*, a resident of the normal flora of the oral cavity, is the main etiologic agent of dental caries [1–3]. Cariogenicity relies on the capacity of the bacterium to: (i) build up biofilm, (ii) acidify the extracellular milieu, and (iii) persist in an adverse environment, three uncommon feasibilities in other bacteria. These capabilities are related to the accumulation of a glycogen-like internal polysaccharide (IPS), whose attributed role is to provide a carbon source during periods of shortage with the concomitant production of acidity [4]. The role of sugars on the pathogenesis of dental biofilm formation determining cariogenicity has been established in good detail [5]; and a direct relationship has been evidenced between the capacity to accumulate IPS and the *in vivo* cariogenic potential of *S*. *mutans* [6]. Besides, the biosynthesis and catabolism of glycogen in prokaryotes have been identified to be critical for virulence and ability of bacteria to build up biofilm [7,8].

The pathways for synthesis of glycogen in prokaryotes and mammals are remarkably different [7–10]. Indeed, the respective enzymes are not homologous and the glucosyl donor used to elongate the α-1,4-glucan is either UDP-Glc (eukaryotes) or ADP-Glc (bacteria). In addition, their regulations are different. In bacteria, the synthesis of ADP-Glc is controlled, but in eukaryotes the regulatory step is the glucan elongation [9]. In prokaryotes, production of ADP-Glc (a metabolite that is not found in mammals) takes place by the reaction catalyzed by ADP-Glc pyrophosphorylase (EC 2.7.7.27; ADP-Glc PPase): ATP + Glc-1P ↔ADP-Glc + PPi. ADP-Glc PPases are enzymes finely regulated by metabolites with the characteristic that, even when varying according to the source, the activator is a key intermediate in the major carbon assimilatory pathway in the respective organism [9,10]. Distinctively from other bacteria, the ADP-Glc PPase from Firmicutes is composed by subunits GlgC and GlgD that give rise different oligomeric forms of the protein [11–13]. This is the case for the enzyme from *S*. *mutans*, which has been recombinantly produced in the GlgC (having low activity), the GlgD (inactive) and the GlgC/GlgD (fully active) forms. GlgC and GlgC/GlgD were found distinctively regulated by metabolites, being the latter (supposedly the functional enzyme found in *S*. *mutans*) inhibited by phospho*enol*pyruvate (PEP) and orthophosphate (Pi), in a way that is overcome by fructose-1,6-bis-phosphate (Fru-1,6-bisP) [11].

Several compounds have been assayed to control the cariogenic process, including many fluoride agents [14]. Among them, sodium monofluorophosphate (MFP) was found to be effective and is thus included in the formulation of toothpastes. Nevertheless, the use of MFP is empirical, as a particular mechanism for its action has never been described. Recently [15], the importance of classical compounds used to treat the caries process has been reviewed and has enumerated several molecules with specific enzymatic targets. Generally, the target enzymes are glycosyltransferases involved in the biosynthesis of exo-polysaccharides for bacterial adhesion and biofilm formation. In this work, we report the inhibitory effect of MFP on the ADP-Glc PPase of *S*. *mutans*, which induces a reduction in IPS biosynthesis and correlates with changes in the physiological features of the microorganism. Because as above detailed, glycogen synthesis takes place by different pathways in mammals and bacteria; we conclude that ADP-Glc PPase represents a key target for MFP to control *S*. *mutans* virulence.

## Methods

### Chemicals

All protein standards, antibiotics, isopropyl—thiogalactoside (IPTG), nalidixic acid and other chemicals were of the highest quality available obtained from Sigma-Aldrich or similar.

### Cultures and *in vivo* assays

*S*. *mutans* ATCC 25175 planktonic cultures were incubated at 37°C in LAPTg medium, (10 g/l yeast extract, 10 g/l trypteine, 15 g/l meat peptone, 10 g/l glucose, 1% v/v Tween 80, pH 6.5) in a 3% CO_2_ atmosphere without stirring. The inoculum consisted of a 12 h culture adjusted to OD_600_ 0.10. The factor for correlating OD_600_ and cellular dry mass (CDW) was determined. All cultures were conducted in triplicate. Acidification was measured using a pH-meter.

The minimal inhibitory concentration (MIC, the lowest compound concentration analyzed that prevents visible growth) for MFP and sodium fluoride (NaF) was determined following the broth and agar dilution method, according to reported protocols [16]. Briefly, serial twofold dilutions of MFP or NaF (in a 0.5–64 mM range) were assayed in planktonic *S*. *mutans* ATCC 25175 cultures. After 24 h, the culture turbidity at OD_600_ was determined to check the growth, which was further confirmed by plating in LAPTg-AGAR (LAPTg medium plus 2% agar).

### Protein methods

The hetero-tetrameric *S*. *mutans* ADP-Glc PPase (the GlgC/GlgD conformation) was recombinantly produced and purified as previously described [11]. The protein concentration was determined by the modified Bradford assay [17] using BSA as a standard.

### Enzyme assays

ADP-Glc PPase was measured following the synthesis of ADP-[^14^C]Glc from [^14^C]Glc1P and ATP, according to reported protocols [18]. The standard reaction mixture contained 100 mM MOPS buffer (pH 8.0), 10 mM MgCl_2_, 1 mM [^14^C]Glc-1P (100–1000 cpm/nmol), 3 mM ATP, 0.5 mU/μl inorganic pyrophosphatase, and 0.2 mg/ml bovine serum albumin plus enzyme in a total volume of 0.2 ml. Reactions were incubated for 10 min at 37°C and terminated by heating in a boiling-water bath for 1 min. The ADP-[^14^C]Glc formed during the reaction was then converted to [^14^C]glycogen by *E*. *coli* glycogen synthase. Then, glycogen was precipitated with 0.1 M KCl (in methanol 75% v/v), washed with the same solution and resuspended in distilled water. Radioactivity was measured by a scintillation counter. One unit (U) of enzyme activity is equal to 1 μmol of product formed per minute under the conditions specified above.

### Calculation of kinetic constants

MFP curves were performed by assaying enzymatic activity at saturating levels of substrates. The experimental data were plotted as relative enzyme activity *versus* MFP concentration, and the kinetic constants were determined by fitting the data to the Hill equation, as described elsewhere [19]. Fitting was performed with the Levenberg-Marquardt nonlinear least-squares algorithm provided by the computer program Origin^™^. The kinetic constant *I*_0.5_ corresponds to the concentration that gives 50% maximal inhibition. All kinetic constants are the mean of at least three sets of reproducible data within ± 10%.

### Extraction and determination of intracellular polysaccharides

Polysaccharide extraction was achieved by a previously described alkali treatment protocol [20–22]. Briefly, samples from *S*. *mutans* cultures were collected by centrifugation, washed with ice-cold water and centrifuged again. Cells were resuspended to OD_600_ 5.0 and boiled for 5 min. Then, 0.3 ml 30% w/v KOH was added per ml of cell suspension, and samples were boiled for 90 min. After cooling, solutions were neutralized with acetic acid, and polysaccharides were precipitated at 0°C with 3 volumes of 97% v/v ethanol. After centrifugation, polysaccharides were dissolved in 0.1 ml water, and a 30 μl aliquot was digested with 2 U of amyloglucosidase from *Aspergillus niger* in 100 mM acetate buffer pH 4.5 for 2 h at 55°C in a final volume of 100 μl. The released glucose was determined by the specific glucose oxidase method [23], and the amount of monosaccharide was taken as a measure of the glycogen content.

## Results

### *In vivo* studies

**Fig 1:**
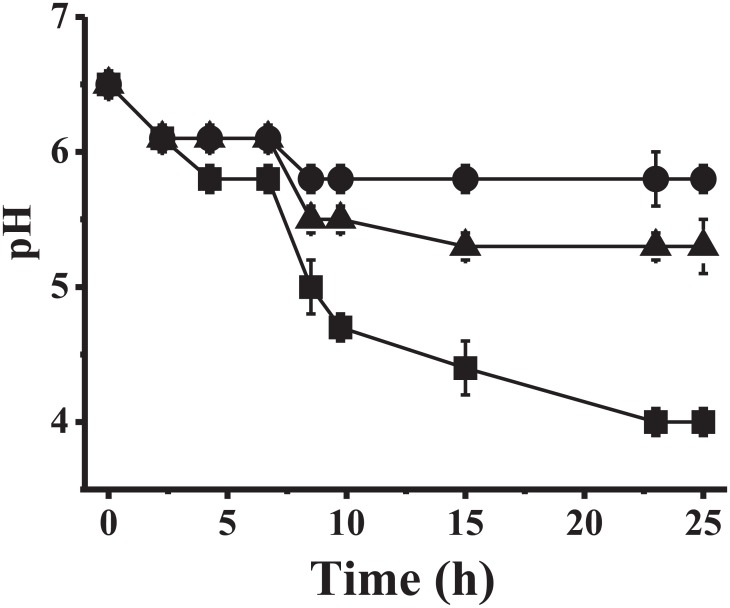
pH reduction from *S*. *mutans* ATCC 25175 planktonic cultures in the presence of 2 mM MFP (circles) or 1 mM NaF (triangles). Cultures with no MFP or NaF are shown in squares.

First, we analyzed the effect of NaF and MFP on the growth of *S*. *mutans* ATCC 25175, determining MIC values of 2 mM and 4 mM, respectively. Taking into account the values of MICs, we decided to analyze the MFP effect by supplementing LAPTg medium with 2 mM MFP as well as by adding 1 mM NaF to establish a comparative analysis, as described below. As shown in Fig 1, a culture of *S*. *mutans* ATCC 25175 lowered the pH of the medium from 6.50 to 4.00 after 24 h (control). On the other hand, when *S*. *mutans* was grown in identical conditions, but with the addition of MFP (2 mM), it lowered the pH of the medium only to 5.29. When 1 mM NaF was added to the control, the pH lowered to 5.80. For this reason, this experiment indicated that both NaF and MFP reduced the acidogenesis ability of the bacterium. In agreement with previous reports, when *S*. *mutans* was grown in the presence of 2 mM Pi, the cells behaved identically to control cultures (data not shown) [24].

**Fig 2:**
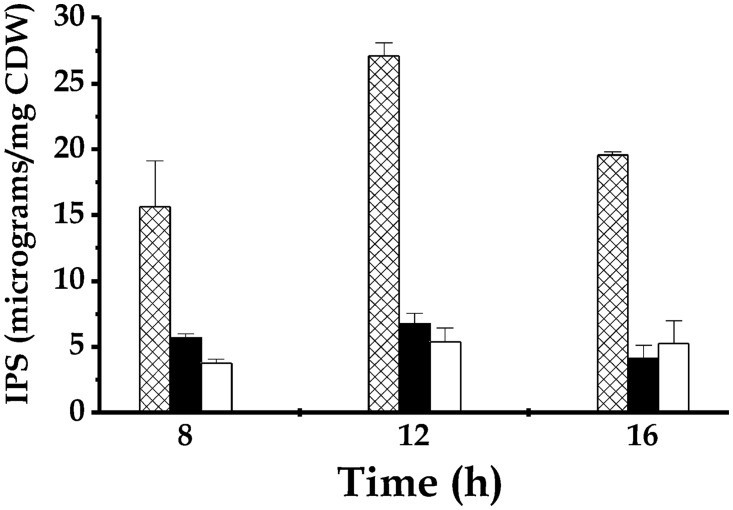
Glycogen content from *S*. *mutans* ATCC 25175 planktonic cultures in the presence of 2 mM MFP (black bars) or 1 mM NaF (white bars). Cultures with no MFP or NaF are shown in plaid bars.

Cultures with and without MFP were analyzed with regard to their IPS content at three points of growth (8, 12 and 16 h). Cultures with NaF were included to compare with previously reported *S*. *mutans* behavior [25]. As shown in Fig 2, IPS accumulation was strongly affected in the presence of 2 mM MFP. Towards the end of the exponential phase (8 h samples), the IPS level was one-third that of the control. Entering the stationary phase (12 h), IPS accumulation was approximately 4-fold lower than control cultures. Additionally, less IPS accumulation was observed in control cultures when the advanced stationary phase was reached. Nevertheless, the ratio (4 to 1) between the IPS content in control and MFP cultures was sustained. In addition, the effect of MFP on IPS accumulation was similar to that exerted by NaF, which is in accordance with previous reports on the effect of NaF on *S*. *mutans* cultures [25]. It is noteworthy that the presence of 2 mM Pi [same concentration as MFP] affected neither growth of *S*. *mutans* cells nor IPS accumulation (data not shown).

### Enzymatic studies

Previously [11], we characterized both active *S*. *mutans* ADP-Glc PPase conformations, GlgC and GlgC/GlgD, and demonstrated that this enzyme catalyzes the key step in IPS synthesis, known to be a major cariogenic factor [4,6,26–28]. In addition, we showed that *S*. *mutans* ADP-Glc PPase activity is allosterically regulated by metabolites from the glycolytic pathway, such as PEP, Fru-1,6-bisP and Pi. Additionally, GlgC/GlgD was sensitive to salt ions, although NaF (up to 25 mM) exhibited no effect [11].

**Fig 3:**
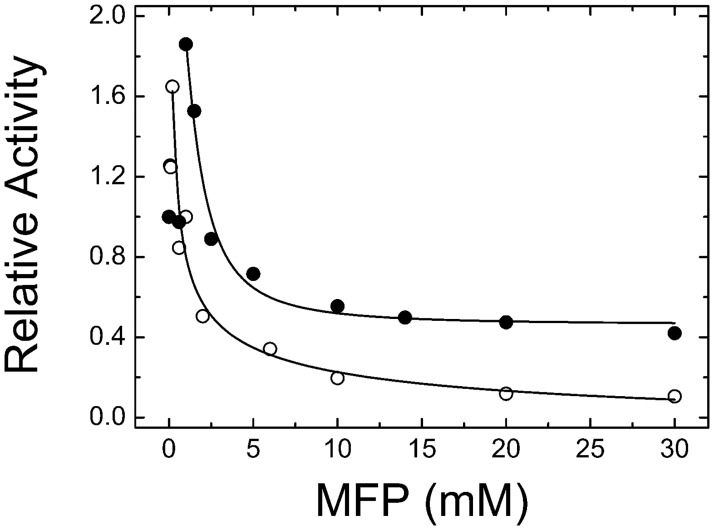
*S*. *mutans* ADP-Glc PPase inhibition when Pi (empty circles) or MFP (filled circles) are present in the reaction mixture. Curves were obtained from reactions conducted at 1 mM Glc-1P, 2 mM ATP and 10 mM Mg^2+^.

**Fig 4:**
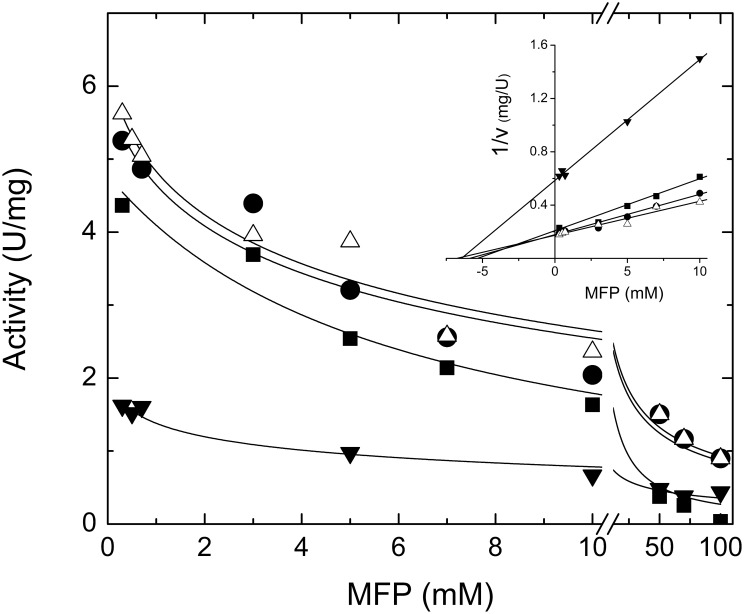
*S*. *mutans* ADP-Glc PPase inhibition curves by MFP at different concentrations (▼-1 mM, ■-3 mM, ●-5 mM and △-10mM) of ATP. In the inset, the data represented according Dixon plots (1/v *vs* MFP concentration) are shown to estimate the inhibition constant.

Because of the structural analogy it exhibits with Pi, we analyzed whether the *S*. *mutans* ADP-Glc PPase is sensitive to MFP. We found that MFP inhibits the hetero-tetrameric GlgC/GlgD (the form with physiologic relevance) enzyme. As it has been reported for the effect of Pi [11], MFP produced somehow complex effect, with a near 2-fold activation at low concentrations followed by a marked inhibitory effect, where enzymatic activity decreased more than 10-fold with an *I*_0.5_ of 1.5 mM (Fig 3). The curves were also conducted at four different ATP concentrations (1, 3, 5 and 10 mM; see Fig 4). As shown, in all the cases the pattern complexity of the effect was similar. Dixon plots [29] of the data obtained in the inhibitory range indicate that the major effect of MFP is not competitive with ATP. Line intercepts suggest that the effect is non-competitive or slightly mixed with an apparent *K*_I_ in the range of 5–7 mM (Fig 4, inset). Fig 4 shows that *I*_0.5_ values for MFP do not strongly depend on ATP concentration. In general, MFP inhibition resembles the effect reported for Pi [11] (see also Fig 3), although the latter has a slightly smaller *I*_0.5_ values. This similarity may be due to the geometry shared by both compounds. However, a difference between the inhibition caused by MFP or Pi is that the latter is slightly reverted by Fru-1,6-bisP, while that of MFP is not (Table 1). In addition, PEP, a highly sensitive inhibitor of GlgC/GlgD (*I*_0.5_ 0.08 mM), is also reverted by Fru-1,6-bisP [11].

**Table 1:** Inhibition of the *S*. *mutans* ADP-Glc PPase activity by Pi or MFP in the absence or presence of Fru-1,6-bisP.

		10 mM Fru-1,6-bisP	1 mM Fru-1,6-bisP	No Fru-1,6-bisP
Inhibitor		Activity (% remaining activity)		
Pi	0 mM	100 ± 8	99 ± 7	100 ± 8
	0.5 mM	67 ± 5	61 ± 7	51 ± 6
	2.5 mM	38 ± 6	31 ± 4	15 ± 2
MFP	0 mM	99 ± 9	99 ± 8	100 ± 9
	5 mM	61 ± 7	65 ± 6	67± 8
	10 mM	42 ± 5	39 ± 5	41 ± 4

In the above context, MFP arises as an inhibitor of IPS synthesis with ADP-Glc PPase as its target, which is the key step in bacterial glycogen synthesis, particularly the cariogenic IPS accumulation in *S*. *mutans*. MFP primarily behaves as an inhibitor of the enzyme because of its analogous structure to the physiological effector Pi, although for the former (contrarily to Pi), the inhibitory effect is not reverted by the regulatory metabolite Fru-1,6-bisP and thus might remain independent of changes in the cellular metabolism. Altogether, the results presented in this work constitute an important mechanistic link for the role of MFP, as *S*. *mutans* with reduced IPS accumulation are less cariogenic and are more quickly removed from the oral microflora [4], opposing those hypercariogenic strains that over-produce IPS [6].

## Discussion

The use of fluoride as an anticariogenic agent has been widely established, and its efficacy involves a complex sum of factors affecting microbial physiology due to the effects of HF or fluoride ions (F^-^) [30]. The latter targets the enzyme enolase (EC 4.2.1.11) in *S*. *mutans* [31,32], decreasing the glycolytic rate and consequently acid production thus diminishing cariogenic effects [30,33]. MFP has been used in toothpaste formulations, but the precise anti-caries mechanism, *i*.*e*., whether MFP itself is an anti-caries agent or whether it is a source of fluoride ion, is not known with certainty [34–36]. Moreover, a specific molecular target for MFP action has never been described and its proven cariostatic effect in clinical trials is comparable to that of NaF. Although there is some uncertainty, it has been suggested that its mode of action stems from MFP being a source of fluoride ions [36].

Results reported herein support the action of MFP through its inhibitory effect on the key enzyme in the biosynthesis of bacterial glycogen. Under comparable conditions, NaF does not exert such an effect, whereby it is shown that the MFP molecule is responsible for inhibition, rather than F^-^ ions that could be generated by hydrolysis. We found that MFP decreases the IPS content of *S*. *mutans*, even in conditions (late exponential phase) where the supply of ATP and Glc-1P (ADP-Glc PPase substrates) are guaranteed. In addition, as we discussed elsewhere, the amount of IPS and acidification capacity correlate with levels of ADP-Glc PPase activity during the exponential growth of *S*. *mutans* [11]. Interestingly we found that *in vitro*, MFP inhibits the key enzyme in polyglucan biosynthesis, a particular feature that distinguishes it from other anticariogenic agents tested, such as NaF. Moreover, the inhibition by MFP exhibits as a critical characteristic that is not reversed by Fru-1,6-bisP, a metabolite indicating sugar utilization; which contrasts with inhibition caused by the physiological inhibitors Pi and PEP (both reversed by Fru-1,6-bisP [11]). In addition, the effect of MFP takes at a metabolic step that is not inhibited by NaF. Many glycosyltransferases were identified as targets for the action of the specific molecules utilized in classical anti-caries compounds [15], although the respective interfering mechanisms (i.e., ligand-protein interaction, effect on kinetics, or others) remain largely unknown. The identification of ADP-Glc PPase as a specific molecular point of inhibition by MFP represents a contribution towards a better understanding of the action of active compounds on the carbohydrate metabolism of oral bacteria. Importantly, illuminating these interactions and specific molecular targets is critical for optimizing and/or designing new treatments for the control of caries.

## Concluding Remarks

MFP affects the capacity of *S*. *mutans* to acidify the medium as well as to accumulate IPS. We found a correlation between *in vivo* and *in vitro* studies, as we determined an enzymatic MFP target (ADP-Glc PPase), thus providing a useful tool for caries control.
